# Formation of Single-Species and Multispecies Biofilm by Isolates from Septic Transfusion Reactions in Platelet Bag Model

**DOI:** 10.3201/eid3009.240372

**Published:** 2024-09

**Authors:** Cheryl Anne Hapip, Erin Fischer, Tamar Perla Feldman, Bethany L. Brown

**Affiliations:** American Red Cross, Rockville, Maryland, USA

**Keywords:** biofilm, platelets for transfusion, septic transfusion reactions, blood safety, bacteria, United States

## Abstract

During 2018–2021, eight septic transfusion reactions occurred from transfusion of platelet units contaminated with *Acinetobacter* spp., *Staphylococcus saprophyticus*, *Leclercia adecarboxylata*, or a combination of those environmental organisms. Whether biofilm formation contributed to evasion of bacterial risk mitigations, including bacterial culture, point-of-care testing, or pathogen-reduction technology, is unclear. We designed a 12-well plate-based method to evaluate environmental determinants of single-species and multispecies biofilm formation in platelets. We evaluated bacteria isolated from septic transfusion reactions for biofilm formation by using crystal violet staining and enumeration of adherent bacteria. Most combinations of bacteria had enhanced biofilm production compared with single bacteria. Combinations involving *L. adecarboxylata* had increased crystal violet biofilm production and adherent bacteria. This study demonstrates that transfusion-relevant bacteria can produce biofilms well together. More work is needed to clarify the effect of biofilms on platelet bacterial risk control strategies, but US Food and Drug Administration–recommended strategies remain acceptable.

Septic transfusion reactions (STRs) from bacterial contamination of platelets are a persistent cause of transfusion-associated deaths. Bacterial risk mitigation strategies are aimed at collection mitigations (e.g., taking donor’s temperature, asking screening questions, and disinfecting skin at venipuncture site), pathogen reduction before storage, and detecting bacterial growth by culture and point-of-care rapid tests (*1*). Despite implementation of US Food and Drug Administration (FDA) guidance on bacterial risk control strategies by blood collection establishments and transfusion services, 8 STRs occurred during 2018–2021 from contaminated platelets units involving *Acinetobacter* spp. alone or in combination with *Staphylococcus saprophyticus*, *Leclercia adecarboxylata*, or both (Table) (*2*–*5*). FDA has communicated heightened awareness around single-species and multispecies contamination of platelet units and noted that the units involved in recent STRs either passed bacterial testing or were pathogen-reduced (*2*,*4*–*6*). Those observations raise the possibility that some bacteria may evade risk mitigations because of the route and timing of contamination or through survival strategies like biofilm production. Genetically related organisms have been isolated in culture bottles (e.g., BacT/ALERT; bioMérieux, https://www.biomerieux.com), on the outside of bags, at a collection set manufacturing facility, and in blood centers, and whole-genome sequencing (WGS) by the Centers for Disease Control and Prevention (CDC) suggests an environmental source of contamination (*3*–*5*).

**Table:** *Acinetobacter* spp. bacteria–related and polymicrobial septic transfusion reactions, United States, May 2018–July 2021*

Year and month	Location (outcome)	Risk mitigation (result)	Bacterial species
2018 May	Northern California	Pathogen-reduction technology	A–S†
2018 May	Utah (fatality)	Aerobic culture (neg)	A
2018 Oct	Connecticut‡	Aerobic culture (neg), rapid antigen test (neg)	A–S
2018 Oct	Connecticut‡	Aerobic culture (neg), rapid antigen test (neg)	A–S
2020 Jun	North and South Carolina (fatality)	Pathogen-reduction technology	A–S–L
2020 Jun	Central Ohio, Pennsylvania, New Jersey	Aerobic culture (neg), anaerobic culture (neg)	A§
2021 Jul	Ohio (fatality)	Pathogen-reduction technology	A–S–L†
2021 Jul	Virginia	Pathogen-reduction technology	S–L
*A, *Acinetobacter* spp.; L, *Leclercia adecarboxylata*; neg, negative; S, *Staphylococcus saprophyticus*. †Clinical isolates used in the experiments. ‡Two separate reactions from a double platelet. §Case was excluded from *Acinetobacter* spp. cluster investigation by Centers for Disease Control and Prevention and US Food and Drug Administration based on whole-genome sequencing data (*5*,*36*).			

Biofilms pose an ongoing challenge to infection control in healthcare settings by protecting bacteria against physical, mechanical, and biochemical methods of cleaning and disinfection, and by shielding bacteria from natural defense and treatments (*7*–*9*). Biofilms are complex structures consisting of single or polymicrobial bacteria and thrive on surfaces with moisture and nutrients. Biofilms initiate detachment of bacterial cells or cluster aggregates, produce endotoxins, have heightened evasion from immune surveillance, and form a protective barrier.

*Acinetobacter* spp., *S. saprophyticus,* and *L. adecarboxylata* can form biofilms (*10*,*11*), although synergistic growth enhancement and relevance of these monomicrobial or polymicrobial biofilms to platelets for transfusion is unknown (*4*,*10*,*12*–*14*). Biofilm-mediated *Acinetobacter* spp. are opportunistic gram-negative pathogens, and infections by those pathogens are an increasingly relevant cause of medical device-related infections, likely because of their ability to rapidly generate resistant factors and tolerate harsh environments (*12*,*15*–*19*).

Environmental conditions, such as blood bag plastics and presence of platelets, may affect biofilm formation and, subsequently, bacterial risk mitigations. Previous laboratory studies have shown robust adhesion of *S. epidermidis* to the internal surface of platelet storage bags in the presence of plasma factors and platelets (*20*–*22*). Further, bacteria exhibit different traits between planktonic and sessile states because bacterial attachment to a surface causes a rapid change in gene expression levels; this mechanism may be important for platelet bag surfaces and other surfaces throughout the supply chain (*9*). Another study conducted using a non–FDA-approved pathogen-reduction technology (Mirasol; TerumoBCT, https://www.terumobct.com) demonstrated that platelet products inoculated with planktonic *S. epidermidis* had ≈1 log fewer bacteria after pathogen reduction than those inoculated with sessile cells, highlighting the potential importance of biofilms formation in platelets (*23*).

*Acinetobacter* spp., *S. saprophyticus*, and *L. adecarboxylata* behavior in coculture and the relevance to platelets for transfusion remains unknown. Preliminary investigations conducted at the American Red Cross Microbiology Laboratory demonstrated the effect of platelets on biofilm matrix production for single and combinations of transfusion-relevant biofilm-producing bacteria (*24*). Our study aimed to address gaps in knowledge by developing a plate-based biofilm evaluation model with platelet-relevant variables using isolates from recent STRs to investigate biofilm formation in contaminated platelets.

## Materials and Methods

### Platelet Products

**Figure 1:**
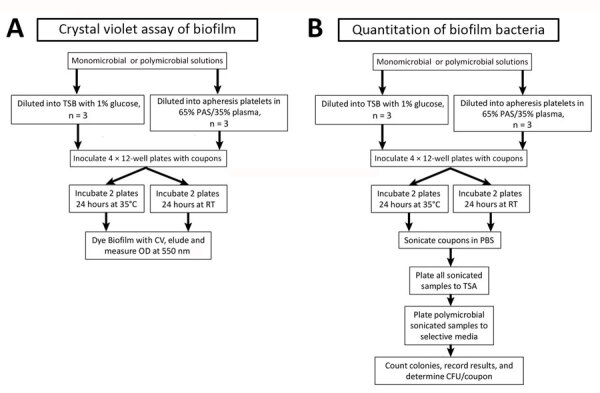
Overview of procedural steps taken to evaluate monomicrobial and polymicrobial biofilms grown on platelet bag coupons in different media (apheresis platelets vs. TSB–glucose) and at 22°C vs. 35°C. A) Study methodology for measuring biofilm production through crystal violet assay. B) Methods for determining CFU per coupon and comparative species composition of polymicrobial biofilms. CV, crystal violet; OD, optical density; PAS, platelet additive solution; RT, room temperature; TSA, tryptic soy agar; TSB, tryptic soy broth.

All platelet donors for this study provided informed consent before collection. We collected platelet units on the Amicus apheresis collection system (Fresenius Kabi, https://www.fresenius-kabi.com) and stored in 65% platelet additive solution (PAS III) (35% plasma). We rested platelets for 2 hours, then agitated them in a platelet incubator overnight at 20°C–24°C. We conducted all experiments with 3–4 independent biologic replicates and performed each biologic replicate with a unique donor collected on a separate day. We tested biologic replicates in technical duplicates (Figure 1).

### Transfusion-Relevant Bacterial Isolates

We obtained bacterial strains of *Acinetobacter* spp. (A), *S*. *saprophyticus* (S), and *L*. *adecarboxylata* (L) from the pathogen-reduced apheresis platelet units involved in the Northern California (May 2018) and the Ohio (July 2021) clinical STR cases (Table). We used a biofilm-producing *S. epidermidis* isolate (ATCC 35984) for the positive control.

### Plastic Platelet Bag Material

We created 15-mm round coupons from Amicus platelet bags (Fresenius Kabi) by using a Cameo 4 instrument (Silhouette, https://www.silhouetteamerica.com). We sterilized the coupons by submerging them in freshly prepared 5% bleach for 5 minutes and then in 70% sterile alcohol for 15 minutes. We aseptically transferred the coupons and washed them in sterile distilled water 3 times to remove excess alcohol (*20*,*22*). We placed the coupons in a sterile 12-well plate (interior side up), which we allowed to air dry in a biosafety cabinet.

### Development of 12-Well Microplate Model to Evaluate Bacterial Biofilms on Platelet Bag Plastics

To standardize testing of environmental variables on biofilm formation in platelet products, we developed a multiwell plate assay that enabled simultaneous evaluation of single and pairwise combinations of bacteria grown on platelet bag plastics. We placed sterilized coupons cut from the platelet storage bags in plate wells before inoculation by using nontreated, sterile 12-well cell culture plates (Wuxi NEST Biotechnology, https://www.nestscientificusa.com). After 24 hours of bacterial growth, we removed the coupons from the plate, washed them, and used them to characterize the adherent biofilm. We used this simplified method to screen for effects of environmental variables on the quantity of viable adherent bacteria, biofilm matrix production, patterns in species composition, and spatial organization on the platelet bag plastic.

### Inoculation and Controls

We transferred bacteria from frozen aliquots onto tryptic soy agar (TSA) plates, streaked them for isolation, and incubated them for 24 hours at 35°C. We added isolated colonies to tryptic soy broth (TSB) and incubated them overnight. We performed dilutions in TSB to obtain a 600-nm optical density (OD) corresponding to a concentration of ≈3 × 10^8^ CFU/mL. A 1:100 dilution of the broth in either apheresis platelets or TSB with glucose (TSBG) gave a final inoculation concentration of ≈3 × 10^6^ CFU/mL. We mixed equal volumes from the single bacterial inoculation tubes to prepare L–S, L–A, A–S, and L–A–S inoculation tubes. We added 1.5 mL from each inoculation tube to each labeled well containing a single coupon for a starting count of ≈4.5 × 10^6^ CFU/well. We set up negative control wells with uninoculated growth medium (platelet or TSBG) with and without coupons. We prepared 2 duplicate sets of plates and incubated them for 24 hours (1 at room temperature and 1 at 35°C).

### Control Checks for Inoculation Suspension Counts, Platelet Toxicity, and Sterility

We confirmed the population counts of the L, A, S, and positive control inoculation suspensions by serially diluting in sterile phosphate-buffered saline (PBS) pH 7.4 and plating the –3 and –4 dilutions by using the spread plate method (100 μL). We kept the inoculation suspension tubes at room temperature for 24 hours and replated them to check for platelet toxicity. We defined platelet toxicity as a countable decrease in bacterial growth.

After 24 hours of incubation, we plated 100 μL from the negative wells onto TSA plates and incubated them at 36°C for 48 hours. We defined sterility as no growth on the TSA plates.

### In Vitro Biofilm Assessment by Crystal Violet Staining Assay

We used crystal violet staining assay as a proxy to indicate the biomass of secreting material with these combinations of clinical isolates from STRs. After 24 hours, we carefully removed the starting inoculum from each well. We gently washed the coupons 2 times with 2 mL PBS. We fixed the coupon biofilms by allowing them to dry on a block heater set at 45°C–50°C for 1 hour. We stained the wells with 1 mL of 0.05% aqueous crystal violet for 15 minutes at room temperature (*22*,*24*). We removed the stain and gently washed the coupons 2 times with 2 mL sterile water (*25*). During the final wash, were removed the coupons and transferred them to new 12-well plates. We used 2 mL 30% (vol/vol) acetic acid to elute the bound crystal violet on the coupon and measured optical density at 550 nm (OD_550_). We performed each assay with duplicate wells for each bacterium (or combination). We subtracted the baseline readings from the coupons containing TSBG and apheresis platelets without bacteria from the readings at OD_550._

### Quantitation of Bacteria within Biofilm

We measured CFUs to quantify the bacteria present in the biofilms formed from these isolates and determine if they grow well together for those cocultured. We washed the wells in PBS and transferred the coupons to sterile Eppendorf tubes containing PBS. We vortexed these tubes thoroughly for 1 minute, placed them in a floating foam rack, and sonicated them at 40 kHz for 30 minutes by using the Branson 5800 Sonicator (Emerson, https://www.emerson.com), (*20*,*21*). After sonication, we thoroughly vortexed the tubes for 1 minute and serially diluted the solution in PBS. We plated dilutions onto TSA in duplicate and the selective and differential media (eosin methylene blue), Leeds, and MSA (mannitol salt agar) plates. We counted colonies on the TSA plates to determine the CFUs per coupon, and we counted the differential plates to determine percentage distribution of bacteria on the mixed coupons.

We quantitated the effect of sonication on the viability of each bacterium by measuring the CFU/mL of a 3 × 10^6^ CFU/per milliliter PBS suspension (by serial dilution) before sonication and after sonication. A decrease in CFU/mL would indicate loss of viability; we noted no loss in viability.

### Microscopic Examination

We washed coupons with biofilm and fixed them with heat by placing the 12-well plates on a block heater set at 45°C–50°C for 1 hour. We Gram-stained the coupons by using a kit (Hardy Diagnostics, https://hardydiagnostics.com) according to the manufacturer’s instructions. We first sonicated duplicate coupons and then the Gram-stained coupons to show efficacy of sonication. We visualized stained coupons by using light microscopic examination with a BX5 series microscope (Olympus, https://www.olympus-global.com) under the 10× objective (100× total magnification) and 60× objective (600× total magnification). We captured images by using Stream Motion Software (Olympus).

### Statistical Analyses

Analyses included 3–4 biologic replicates per condition (Figure 1). Each biologic replicate represented a unique platelet donor tested in an independent experiment. We performed statistical analysis and visualization by using Excel version 2405 (Microsoft, https://www.microsoft.com) and Prism version 10 (GraphPad, https://www.graphpad.com). We identified statistical differences by using 2-way analysis of variance (ANOVA) or by application of a mixed effects model. We considered a p value of <0.05 as significant. We used Šídák’s multiple comparison test to test the effect of a condition on an individual bacterial species when ANOVA or a mixed-effects model yielded statistically significant results. We used a threshold of p<0.05 to determine significance of the adjusted p value (p_adj_), which we determined by using multiple comparison testing.

## Results

### Effect of Growth Medium on Biofilm Formation on Platelet Bag Plastic

**Figure 2:**
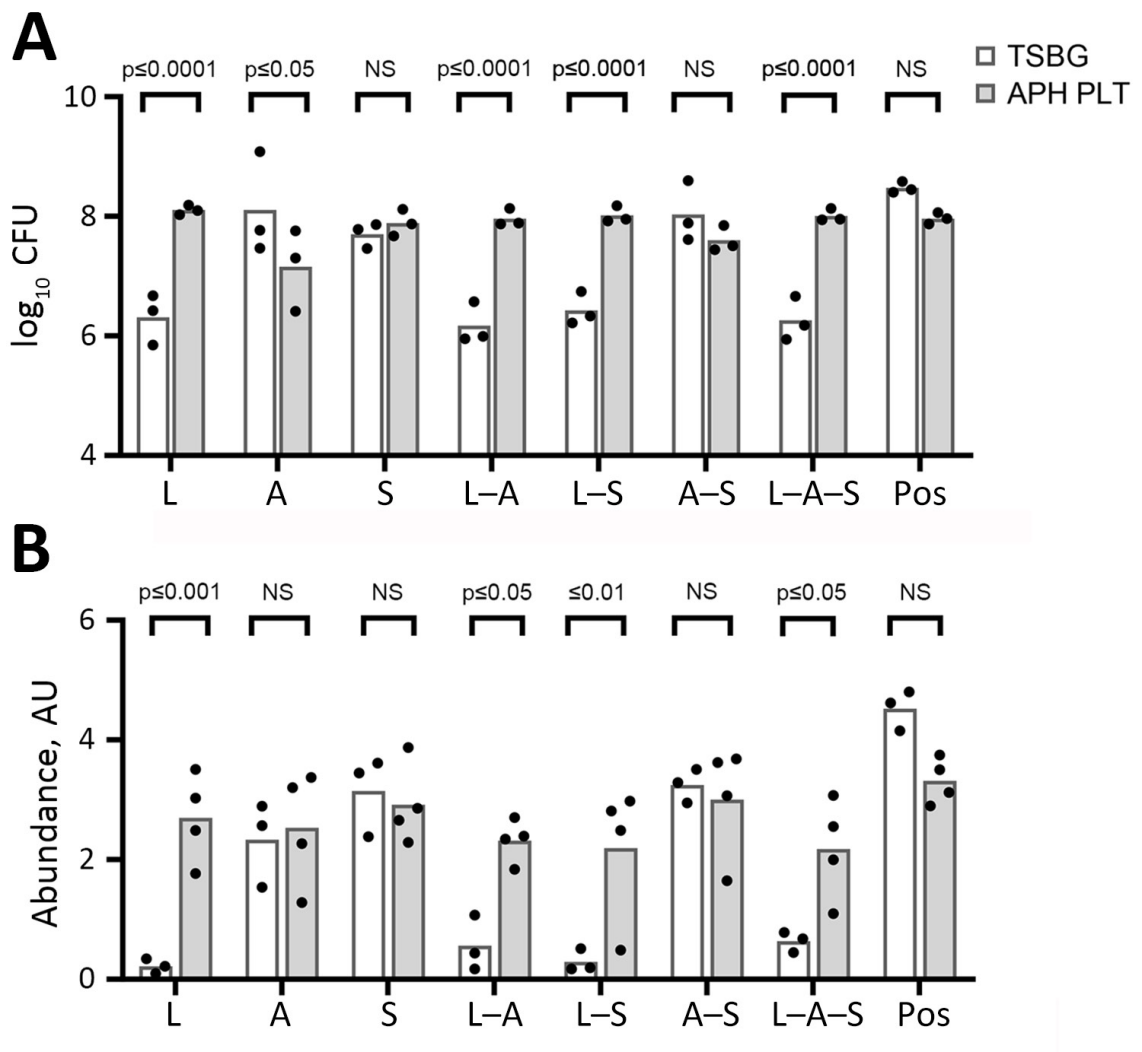
Effects of different growth media (TSBG) versus APH PLT for most bacteria on biofilm CFU recovery (A) and CV biofilm formation (B) on platelet bag coupons after 24 hours of incubation at 35°C. Baseline readings of TSBG and APH PLT without bacteria were subtracted from the readings at OD_550._ *Leclercia adecarboxylata* and the polymicrobial biofilms containing *L. adecarboxylata* showed a significant (p<0.05) increase in CFU within the biofilm and CV biomass when grown in APH PLT compared with TSBG. *Acinetobacter* spp. recovered from the biofilm was significantly decreased when grown in APH PLT. Each dot represents an individual biologic replicate inoculated with the corresponding monomicrobial or polymicrobial bacterial species. A, *Acinetobacter* spp.; APH PLT, apheresis platelets; AU, absorbance unit; CV, crystal violet; L, *L. adecarboxylata*; NS, not significant; OD, optical density; pos, positive control (*Staphylococcus epidermidis*); S, *S. saprophyticus*; TSBG, tryptic soy broth–glucose.

We investigated the effect of growth medium on bacterial burden and biofilm matrix production on storage bag plastic for all single and pairwise combinations of A, S, and L. By using our multiwell plate assay, we grew bacteria at 35°C in TSBG liquid medium or in apheresis platelets in PAS III (APH PLTs) from 3 unique donors. We found that all bacterial monoculture or coculture combinations in TSBG or APH PLTs resulted in viable bacteria adhered to the platelet storage bag plastic (Figure 2, panel A). The choice of growth medium had a differential effect depending on the bacterial species (or combination). Total CFU counts of adherent bacteria were higher when L (p_adj_<0.0001), L–S (p_adj_<0.0001), and L–A–S (p_adj_ = 0.0058) were grown in APH PLTs compared with TSBG. In contrast, growth in APH PLTs appeared to diminish the number of bacteria adhered to the plastic coupon in monoculture of *Acinetobacter* alone by a log (p_adj_ = 0.0283). In a separate set of experiments, we used a crystal violet staining assay to quantify the production of biofilm matrix on the platelet storage bag coupon when bacteria were grown in TSBG compared with APH PLTs (Figure 2, panel B). Growth in APH PLTs resulted in a mean 12-fold increase in crystal violet staining of platelet plastic in cultures inoculated with *L. adecarboxylata* alone (p_adj_ = 0.0002) and mean 4.1-fold, 7.5-fold, and 3.4-fold increases when *L. adecarboxylata* was cultured in combination with *Acinetobacter* spp. (p_adj_ = 0.0123), *S. saprophyticus* (p_adj_ = 0.0056), or both *Acinetobacter* spp. and *S. saprophyticus* (p_adj_ = 0.038).

### Limited Effect of Temperature on Biofilm Formation by STR Isolates

**Figure 3:**
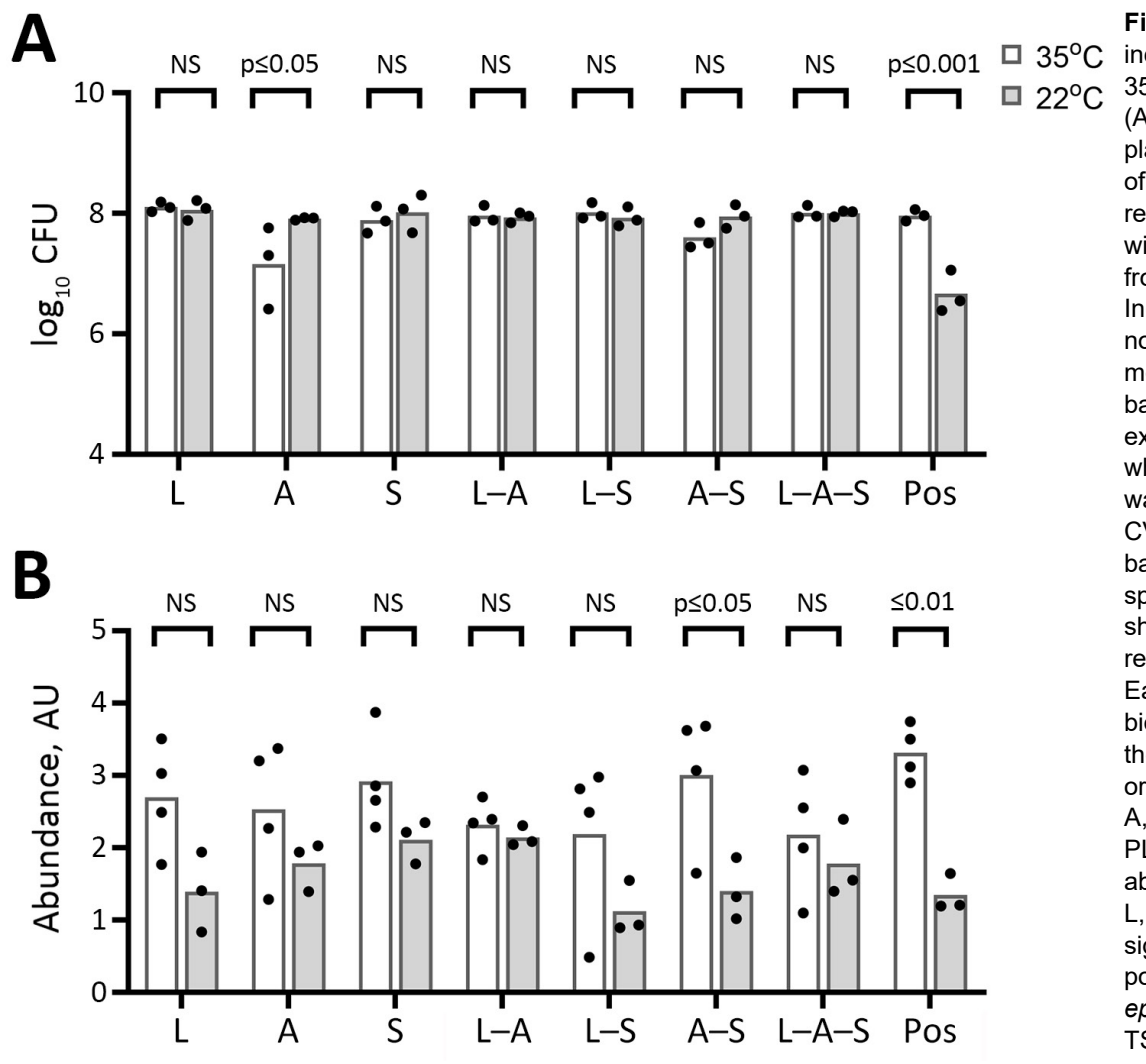
Effects of different incubation temperatures (22°C vs. 35°C) on biofilm CFU recovered (A) and CV biofilm formation (B) on platelet bag coupons after 24 hours of incubation in APH PLT. Baseline readings of TSBG and APH PLT with no bacteria were subtracted from the readings at OD_550._ Incubation temperature showed no significant effect on quantitative monomicrobial or polymicrobial bacterial growth in APH PLTs, except for *Acinetobacter* spp. alone, which was lower at 35°C. There was a trend of decreased bound CV at room temperature for all bacteria, but only the *Acinetobacter* spp./*S. saprophyticus* combination showed statistically significant reduction between 35°C and 22°C. Each dot represents an individual biologic replicate inoculated with the corresponding monomicrobial or polymicrobial bacterial species. A, *Acinetobacter spp.*; APH PLT, apheresis platelets; AU, absorbance unit; CV, crystal violet; L, *Leclercia adecarboxylata*; NS, not significant; OD, optical density; pos, positive control (*Staphylococcus epidermidis*); S, *S. saprophyticus*; TSBG, tryptic soy broth–glucose.

Many environmental bacteria thrive at 22°C, which is the temperature for conventional platelet storage. Therefore, we evaluated biofilm formation in APH PLTs at 22°C. Apart from *Acinetobacter* spp. alone, we did not detect a significant effect of growth in APH PLTs at 22°C on the number of STR bacteria adhered to the platelet plastic coupons compared with 35°C (Figure 3, panel A). When *Acinetobacter* spp. was cultured alone at 22°C, we found a mean log increase of 0.75 compared with growth at 35°C (p_adj_ = 0.0228). Although few significant differences were found in biofilm matrix production at 22°C compared with 35°C, we did observe a trend toward a decrease of bound crystal violet in biofilm matrix or biomass at room temperature (Figure 3, panel B).

### *L. adecarboxylata* Compared with Other STR Bacteria in Multispecies Biofilms

**Figure 4:**
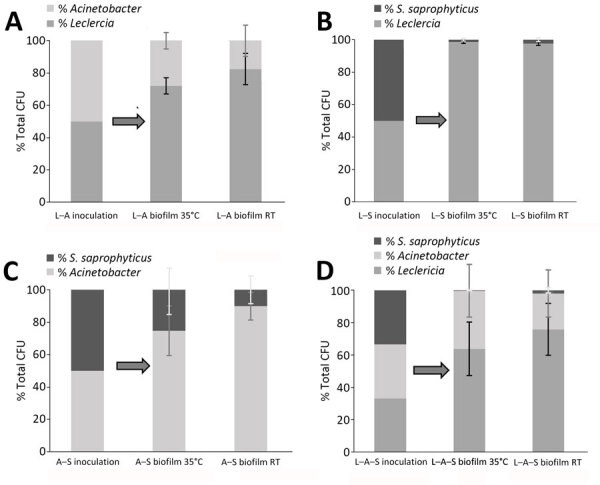
Average total CFU (n = 3) percentage comparison for 4 combinations of *Leclercia adecarboxylata*, *Acinetobacter* spp., and *Staphylococcus saprophyticus* mixed in equal parts and incubated together. Each bacterial species was measured in each polymicrobial biofilm after 24-hour incubation in apheresis platelets at both 35°C and room temperature. A) *L. adecarboxylata* and *Acinetobacter* spp.; B) *L. adecarboxylata* and *S. saprophyticus*; C) *Acinetobacter* spp. and *S. saprophyticus;* D) all 3 species incubated together. Even with a longer doubling time, *L. adecarboxylata* outcompeted *Acinetobacter* spp. in CFU percentage, whereas *S. saprophyticus* only accounts for 2% CFU in L–A–S polymicrobial biofilm grown at room temperature. Error bars represent SDs of the replicates. A, *Acinetobacter spp.*; L, *L. adecarboxylata*; RT, room temperature; S, *S. saprophyticus*.

To better understand the composition of multispecies biofilms, we used selective and differential media to quantify the contribution of each species to the overall number of viable, adherent bacteria on the platelet bag coupons (Figure 4). The inoculum for cocultures of bacteria in APH PLTs was composed of equal proportions of either pairs or all 3 of the STR-relevant bacterial species. Regardless of incubation temperature, we found that *L. adecarboxylata* made up the highest percentage of the total CFUs on the platelet bag coupon when grown in combination with *Acinetobacter* spp., *S. saprophyticus*, or both. The population of *S. saprophyticus* was limited in the multispecies biofilms compared with *Acinetobacter* spp. and *L. adecarboxylata*. When all 3 species were cultured together, *S. saprophyticus* made up no more than 2% of the total CFU counts of bacteria adhered to the plastic coupon.

When grown in APH PLT or PAS III in 22°C conditions, the doubling rate for *S. saprophyticus* isolate is ≈169 minutes in the first 24 hours (Erin Fischer, American Red Cross unpub. data). This slower doubling rate, in comparison to *L. adecarboxylata* at 113 minutes and *Acinetobacter* spp. at 88 minutes, could contribute to the low total CFU percentage in the mixed biofilm. However, even the faster doubling rate of *Acinetobacter* spp. does not outcompete *L. adecarboxylata* in a polymicrobial biofilm incubated at either 22°C or 35°C (Figure 4).

### Efficacy of Sonication

**Figure 5:**
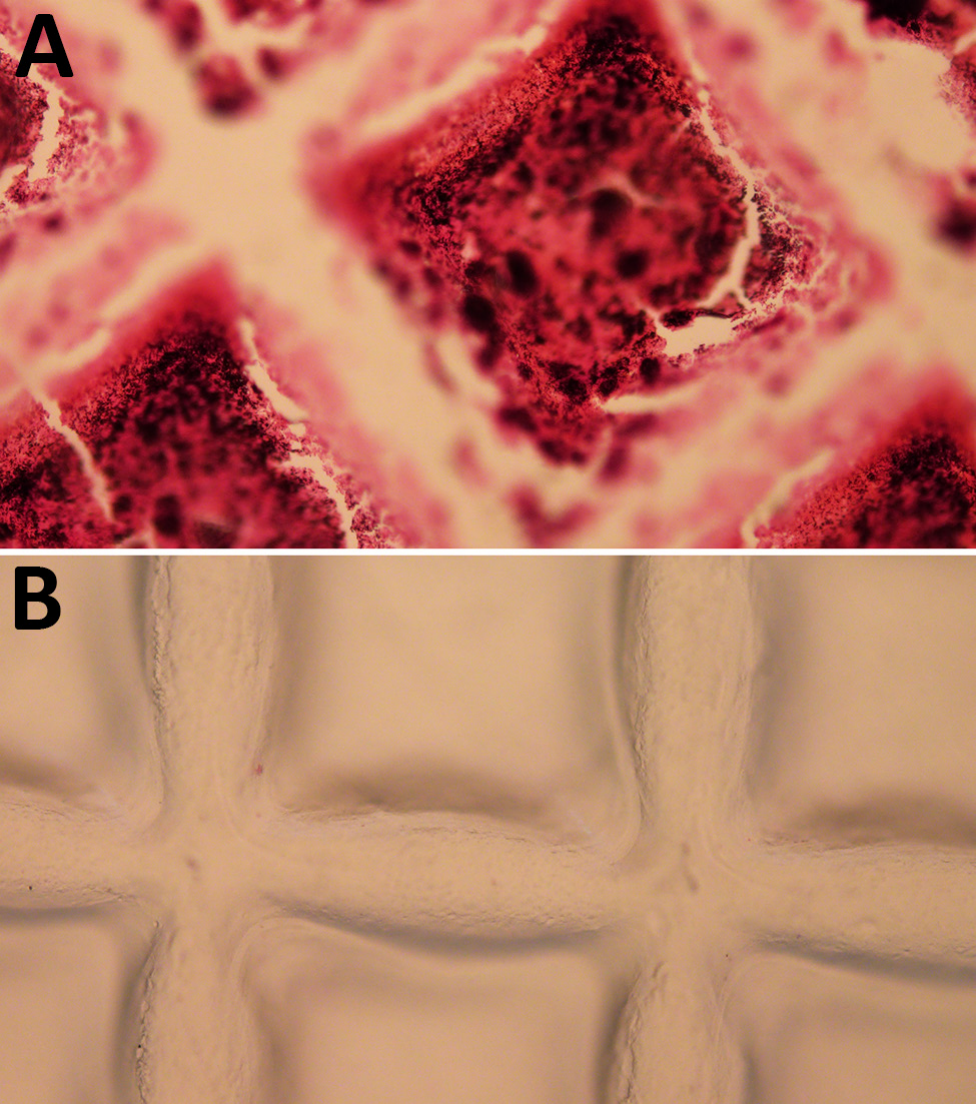
Gram-stained platelet bag coupons incubated with *Staphylococcus saprophyticus* in APH PLT before sonication and Gram-stained duplicate coupons after sonication. A) Accumulation of platelets and gram-positive cocci on the coupon. B) Visual analysis after sonication showing no evidence of platelets or cocci on the surface of the coupon, indicating that the sonication procedure effectively removed the biofilm and bacteria. The grid pattern observed on the coupon reflects the texture of the inner surface of Amicus (Fresenius Kabi, https://www.fresenius-kabi.com) platelet bags. APH PLT, apheresis platelets.

Disruption and bacterial enumeration of *Staphylococcus* spp. forming biofilms by sonication has received variable results (*26*). We incubated duplicate coupons in *S. saprophyticus*–inoculated APH PLT following our quantitation of biofilm bacteria study design (Figure 1). We Gram-stained coupons before the sonication stage and Gram-stained duplicate coupons after sonication (Figure 5). We examined the accumulation of platelets and gram-positive cocci on the coupon (Figure 5, panel A) and noted no evidence of platelets or cocci on the surface of the coupon after sonication (Figure 5, panel B), demonstrating that the sonication procedure was effective at removing the biofilm and bacteria from platelet coupons.

## Discussion

In an investigation of the STRs that occurred during May–October 2018, CDC and FDA demonstrated that a subset of *Acinetobacter* spp. isolates from patients in 3 different states belonged to a novel taxon of *A. calcoaceticus–baumannii* complex (*3*). Later, Kracalik et al. (*4*) and Villa et al. (*5*) provided an update to the collaborative CDC and FDA investigations to include WGS data that suggested a common environmental source of bacteria upstream of blood manufacturing (*4*,*5*). Kracalik et al. (*4*) recommended additional studies to elucidate the effects of biofilm development on platelet bacterial risk control strategies.

Our goal was to investigate biofilm formation in platelet products by using specific isolates of *Acinetobacter* spp., *S. saprophyticus*, and *L. adecarboxylata* implicated in the 2018–2021 STR cases. Our study demonstrates that transfusion-relevant bacteria can produce biofilms well in monoculture and coculture combinations and that environmental conditions, such as growth medium and plastics, affect biofilms.

Our data showed a limited effect of temperature on biofilm formation by the STR isolates and that growth medium had a more significant effect on growth. As expected, biomass in APH PLTs was significantly higher than in TSBG. We observed a mean 12-fold increase in crystal violet staining of platelet plastic in cultures inoculated with *L. adecarboxylata* alone and mean 4.1-fold and 7.5-fold increases when *L. adecarboxylata* was cultured in combination with *Acinetobacter* spp. or *S. saprophyticus*. *L. adecarboxylata* also showed a significant increase in CFUs within the biofilm when grown in APH PLT compared with TSBG. Conversely, *Acinetobacter* spp. recovered from the biofilm significantly decreased when grown in APH PLT. Of note, our data also demonstrated that *L. adecarboxylata* outcompetes both *S. saprophyticus* and *Acinetobacter* spp. in a polymicrobial biofilm incubated in APH PLTs at either 22°C or 35°C.

Biofilm formation and composition changes with the environment, as previously shown (*27*,*28*). Our results are in line with previous reports that the platelet storage environment affects bacterial growth (*21*,*29*). Other research has shown that growing bacteria in the presence of platelets induces changes in expression of genes associated with biofilm maturation (*30*). Gene expression is controlled by many external and internal factors, such as 2-component or multicomponent signal transduction systems, quorum-sensing, small RNA, and secondary messengers such as cAMP; those systems monitor the environment and regulate the production of exopolysaccharides, fibrins, lipoproteins, and surface-associated proteins (pili and flagella), which together make up the biofilm biomass. Although our study does not address gene expression, the recent publication of WGS results for the STR isolates by CDC and FDA will enable future studies to address the role of specific genes in evasion of bacterial risk control strategies. The effect of platelets on expression of bacterial virulence factors remains poorly understood for bacteria that are classically associated with platelet contamination. Whether platelets and other variables in the platelet storage environment promote biofilm formation universally is an open question that warrants further exploration.

One limitations of our study is that the assays used were plate-based; therefore, we did not consider the effect of gas exchange and agitation in the platelet storage environment. To stain the biofilms, we used crystal violet, which is a basic aniline dye that binds to negatively charged peptidoglycan, DNA, extracellular protein, and polysaccharides. Bacterial gene expression in platelets is changed during growth in platelets compared with TSBG (*21*), and this change would most likely result in biofilm composition variations with differences in crystal violet–bound negatively charged molecules. Variation in biofilm production and composition also poses a challenge for selecting a bacterial strain that does not produce biofilm under all conditions tested in this study. Other research has demonstrated that strains thought to be biofilm-negative will produce biofilm under platelet storage conditions (*29*). Improved genotypic characterization of STR isolates could provide an avenue for engineering a biofilm-negative strain in future studies. Further, we did not conduct confocal microscopic examination, which limits our view of the formation, development, morphology, and structure of the biofilms.

Previous studies have proposed that some biofilm-forming bacteria may evade detection by bacterial culture or pathogen reduction and that planktonic versus sessile cells can affect efficacy (*4*,*23*,*31*). Phylogenetic evidence presented by the CDC and FDA investigation demonstrates genetic relatedness between the STR strains and isolates collected at the platelet collection set manufacturing site, strongly supporting the hypothesis that these bacteria were initially introduced into blood collection establishments from an upstream source. Of note, related strains also have been isolated from the hospital and blood bank environment, and some studies have noted microscopic bag leaks and variability in cocultured isolates implicated in these *Acinetobacter*-related STR (*32*). The relative contribution of limit of detection and low bacterial load, presence of inactivated pathogens, and biofilm formation or other evasion strategies to the failure of bacterial risk mitigation strategies is unknown. If biofilm formation causes evasion of bacterial testing or pathogen reduction, several questions remain for further investigation, including whether biofilms protect bacteria from the psoralen or UV light penetration needed for inactivation, whether viable but nonculturable cells found in some bacterial biofilms are immune to the pathogen-reduction process, and whether certain parts of the platelet bag are more susceptible to biofilm formation than others.

In future studies on the relevance of biofilms in platelets for transfusion, using the correct genetic background when studying bacteria will be key. Researchers might consider reviewing the World Health Organization repository for platelet STR strains, particularly with the novel *A. calcoaceticus–baumannii* clusters identified in these recent STR cases and subsequent CDC and FDA investigations (*33*). Some species demonstrate that biofilm phenotypes differ between laboratory-adapted reference strains and clinical isolates, but whether this phylogenetic distinction translates to phenotypic differences is unknown (*34*). It may also be of interest to investigate bacterial contamination of indwelling catheters used to infuse blood products and to better understand bacterial load that may cause STRs given that, in some cases, co-components of platelets involved in STRs have been transfused without implication (*5*).

The FDA bacterial risk mitigation strategies remain acceptable, and more work is needed to understand the gaps in information regarding the 2018–2021 *Acinetobacter*-related STRs in the United States (*5*,*35*). Given the emerging medical device–related biofilm risks, a need exists to clarify the effects of biofilms on bacterial risk mitigation strategies and for innovative technologies to manage the complexities presented by biofilms (*9*).
